# Amphiphilic and Perfluorinated Poly(3-Hydroxyalkanoate) Nanocapsules for ^19^F Magnetic Resonance Imaging

**DOI:** 10.3390/bioengineering8090121

**Published:** 2021-09-09

**Authors:** Marion Le Gal, Estelle Renard, Christelle Simon-Colin, Benoit Larrat, Valérie Langlois

**Affiliations:** 1 Laboratoire de Microbiologie des Environnements Extrêmes, CNRS, Ifremer, University Brest, F-29280 Plouzané, France; mlegal@ifremer.fr (M.L.G.); christelle.simon.colin@ifremer.fr (C.S.-C.); 2 ICMPE, CNRS, University Paris Est Creteil, F-94010 Creteil, France; renard@icmpe.cnrs.fr; 3 Université Paris-Saclay, CEA, CNRS, NeuroSpin, 91191 Gif-sur-Yvette, France; benoit.larrat@cea.fr

**Keywords:** polyhydroxyalkanoates, mcl-PHA, nanocapsules, fluorinated polymer, amphiphilic copolymer

## Abstract

Nanoparticles have recently emerged as valuable tools in biomedical imaging techniques. Here PEGylated and fluorinated nanocapsules based on poly(3-hydroxyalkanoate) containing a liquid core of perfluorooctyl bromide PFOB were formulated by an emulsion-evaporation process as potential ^19^F MRI imaging agents. Unsaturated poly(hydroxyalkanoate), PHAU, was produced by marine bacteria using coprah oil and undecenoic acid as substrates. PHA-g-(F; PEG) was prepared by two successive controlled thiol-ene reactions from PHAU with firstly three fluorinated thiols having from 3 up to 17 fluorine atoms and secondly with PEG-SH. The resulting PHA-g-(F; PEG)-based PFOB nanocapsules, with a diameter close to 250–300 nm, are shown to be visible in ^19^F MRI with an acquisition time of 15 min. The results showed that PFOB-nanocapsules based on PHA-g-(F; PEG) have the potential to be used as novel contrast agents for ^19^F MRI.

## 1. Introduction

^19^F magnetic resonance imaging (MRI) has considerably developed in recent years in clinical practice [1,2,3,4]. The most frequently used contrast agents are liquid perfluorocarbons, such as perfluoro-15-crown-5-ether, perfluorooctyl bromide (PFOB) (17 fluorine atoms) and perfluoropolyethers (PFPE) [5,6,7]. These molecules have intrinsic physico-chemical and biological properties that are particularly well suited for in vivo applications, such as non-toxicity and biological and chemical inertness [8,9,10,11]. Due to their hydrophobic and lipophobic character, they are formulated with surfactants or encapsulated in polymer particles [12,13]. Thus, several surfactants have been studied to ensure capsule formation with a PLGA polymer shell and a PFOB liquid core such as polyvinyl alcohol and sodium cholate [14]. Systemic administration of these systems requires coatings to prevent uptake by macrophages. For this, different strategies have been developed including surface coating with polysaccharides or polyethylene glycol (PEG) chains [15]. PEGylated polyester nanocapsules have been shown to be of interest for MRI and their long circulating properties and capacity to diagnose tumors have been demonstrated in vivo [15,16]. Furthermore, theranostic capabilities to observe drug accumulation and release from a PFOB-loaded nanocarrier have been shown [17,18,19]. Fluorinated copolymers containing fluorinated units based on acrylic acid derivatives as 2,2,2-trifluorethylacrylate [20], 2,2,2-trifluorethyl methacrylate [21,22], poly[N-(2,2-difluorethyl) acrylamide] [23] or octafluoropentyl methacrylate [24] were synthesized. Although a high density of fluorine atoms is required to be MRI visible [25,26,27], it may lead to an aggregation in aqueous medium because of the hydrophobic character of the fluorinated groups. Moreover, Houvenagel et al. showed that the presence of fluorinated groups into the polymer itself considerably improves the encapsulation rate of PFOB inside the nanoparticles compared to the native polymer [28]. In order to increase the stability in water, hydrophilic blocks were added. Fluoro-copolymers exhibit self-assembly properties [28,29,30,31], which rely on the ability of fluorinated molecules to assembly into a fluorinated phase that is both hydrophobic and lipophobic [32,33,34].

In this context, we propose to design a novel polymeric amphiphilic probe suitable for ^19^F MRI that possesses high fluorine atom content, adequate water solubility and reliable biodegradability property. Poly(3-hydroxyalkanoates) (PHAs) are natural polyesters produced and accumulated by many bacteria as carbon and energy supply when essential nutrients are limited [35,36,37]. Biomaterials based on PHAs have been recently developed for medical or pharmaceutical applications such as tissue engineering, implants or drug delivery carriers due to their proven biodegradability, biocompatibility and lack of toxicity [38,39,40,41]. The combinations of the hydrophobic character of PHA and hydrophilic PEG were used to prepare amphiphilic block and graft copolymers [42]. The grafting reactions are mainly realized on unsaturated PHAs. The most common unsaturated PHA obtained by fermentation [43] in presence of 10-undecenoïc acid is the poly(3-hydroxyoctanoate-co-3-hydroxyundecenoate) which contains terminal alkene groups in the side chains. Water soluble poly(3-hydroxyalkanoate) containing ionic groups was recently designed by two successive photo-activated thiol-ene reactions in presence of sodium-3-mercapto-1-ethanesulfonate and PEG methyl ether thiol to both introduce ionic groups and hydrophilic block [44]. Babinot previously developed multicompartment micelles (MCMs) based on grafted PHAs. Thiol-ene addition is used to graft sequentially perfluorooctyl chains and PEG oligomers onto poly(3-hydroxyoctanoate-co-hydroxyundecenoate) oligomers [45].

Herein, we report on the design of amphiphilic fluorinated PHA using a straightforward methodology starting from a novel unsaturated mcl-PHA, (PHAU). PHAU, produced by marine bacteria *Pseudomonas raguenesii*, is a statistical copolymer bearing pendant hydrophobic unsaturated chains. Our strategy relies on a stepwise use of the thiol-ene addition to graft perfluorinated chains containing from 3 to 17 fluorine atoms and hydrophilic poly(ethylene glycol) (PEG) chains onto PHAU unsaturated side chains. Nanocapsules containing a liquid core of perfluorooctyl bromide (PFOB) were prepared by an emulsion-evaporation process and designed as contrast agents for ^19^F MRI.

## 2. Materials and Methods

### 2.1. Materials 

Chloroform (CHCl_3_), methanol (MeOH) and dichloromethane (CH_2_Cl_2_) were obtained from Carlo Erba (Val de Reuil, France). Dialysis membranes (MWCO 1 and 6–8 KDa) were procured from Spectrumlabs. Polyethylene glycol 550 monomethyl ether and 4-toluenesulfonyl chloride (TsCl) were obtained from Fluka (Buchs, Switzerland), potassium thioacetate and 4-dimethylamino pyridine (DMAP) were purchased from Sigma Aldrich (Burlington, MA, USA). Perfluorodecan-1-thiol was purchased from ELF-Atochem (Colombes, France), perfluorooctan-1-thiol and perfluorooctyl bromide from Sigma Aldrich (Burlington, MA, USA) and trifluoropropan-1-thiol from Fluorochem (Glossop, UK).

### 2.2. PHA_(72)_U_(28)_ Biosynthesis

*Pseudomonas raguenesii* sp. nov. (strain CNCM I-4063 in the Collection Nationale de Cultures de Microorganismes, Institut Pasteur, Paris, France) [46] was cultivated in a two-steps batch cultivation process. In the first step, the cells were inoculated at 10% (*v*/*v*) with a suspension of cells in exponential phase and grown in 5 L Erlenmeyer flasks containing 2 L of marine broth medium (for 1 L: 5 g peptone, 1 g yeast extract, 20 g Sea Salts, pH 7.6) in an incubator at 34 °C, 160 rpm. After the cultivation for 8 h, cells were harvested by centrifugation (7000 rpm, 15 min) and transferred into 30 L fermenter containing 18 L nitrogen free medium (15 g.L^−1^ sea salts) enriched with a mixture of coprah oil (5 g.L^−1^) and 10-undecenoic acid (0.6 g.L^−1^). Culture was incubated at 34 °C, pH is adjusted at 7.6 throughout the fermentation process by automatic addition of 1M NaOH or 1M H_2_SO_4_ and the oxygenation is ensured by a light air flow. Following cultivation for 72 h, cells were harvested by centrifugation (7000 rpm for 15 min) and the pellets lyophilized prior to PHA extraction. Pellets of freeze-dried cells were ground with a mortar and pestle; the resulting powder was extracted with chloroform for 4 h at 50 °C. The PHA-containing chloroform phase was washed once with water and concentrated. The organic phase was evaporated to dryness, and purified PHA were obtained by repeated precipitations in 10 volumes of cold methanol.

### 2.3. PEG-SH Synthesis

PEG-SH was synthesized according to a method described elsewhere [47] carried out in three steps. Tosylation of PEG_550_ was carried out by mixing 30 g of MeO-PEG_550_ (54.5 mmol) and 33 mg of 4-dimethylamino pyridine (DMAP) (2.7 mmol) dissolved in 160 mL of CH_2_Cl_2_/pyridine (1:1) solution at 0 °C under argon atmosphere. Furthermore, 18.7 g of 4-toluenesulfonyl chloride (TsCl) (98.1 mmol) was dissolved in 50 mL of CH_2_Cl_2_ and added dropwise and the solution was stirred overnight until brought back at RT. Then, 200 mL of cold water were added and the aqueous phase was extracted 3 times with 150 mL of CH_2_Cl_2_. The accumulated organic phases were washed successively with 3 × 250 mL of a 1M HCl solution and 3 × 250 mL of a saturated NaCl solution. The solution obtained was dried over MgSO_4_ and then concentrated under reduced pressure to obtain 37.2 g of product (PEG_550_-OTs). To carry out the synthesis of PEG_550_-thioacetate, 37.2 g of PEG_550_-OTs (53 mmol) was dissolved in 200 mL of THF under argon. Then, 11.6 g of potassium thioacetate (102 mmol) was added and the solution was refluxed for 20 h, inducing the formation of a white precipitate. After returning to RT, 400 mL of water were added. Contents of the flask were extracted with 3 × 150 mL of CH_2_Cl_2_ and the organic phase was washed with 3 × 200 mL of water, treated with activated carbon, filtered and evaporated until an oil was obtained (15.3 g, yield 36%).

To obtain the final PEG_550_-SH, 15.3 g of PEG_550_-thioacetate (25 mmol) was dissolved in 200 mL of anhydrous MeOH under argon. Then 15 mL of 37% HCl were added and the resulting solution was refluxed for 2 h. Furthermore, 200 mL of water were added and the solution was extracted with 3 × 150 mL of CH_2_Cl_2_. The combined organic phases were washed with 3 × 200 of water and 3 × 250 mL of NaCl solution, dried over MgSO_4_ and filtered. The solution was evaporated until a PEG_550_-SH oil was obtained (11 g, yield 77%).

### 2.4. Synthesis of PHAU-g-(F) 

Then 200 mg of PHA_(72)_U_(28)_ (1233 × 10^5^ g.mol^−1^, 3.54 × 10^−4^ mol C=C), 29 mg of AIBN (0.5 neq, 1.77 × 10^−4^ mol) and the necessary quantity of fluorinated thiol to obtain 0.8 neq corresponding to 135.8 mg of perfluorodecan-1-thiol (480 g.mol^−1^), 107.6 mg of perfluooroctyl mercaptan (380 g.mol^−1^), 37 mg of trifluoropropyl mercaptan (130 g.mol^−1^) were dissolved in 6 mL of anhydrous toluene under argon, and solutions were heated at 80 °C for 20 h. Toluene was rotary evaporated and ^1^H NMR was conducted to determine the amount of fluorinated compounds grafted.

### 2.5. Synthesis of PHA-g-(F; PEG) 

Next, 200 mg of PHA previously grafted with fluorinated molecule, AIBN and PEG-SH were dissolved in 6 mL of anhydrous toluene under argon in the following proportions: for PHA_(72)_C_8_F_17(12)_PEG_(16)_ 170 mg of PEG-SH, for PHA_(72)_U_(20)_C_6_F_13(8)_ 230 mg of PEG-SH and for PHA_(72)_U_(20)_CF_3(8)_ 242 mg of PEG-SH were added. Solutions were heated at 80 °C for 20 h. Toluene was rotary evaporated and grafted polymers were dissolved in 30 mL of acetone. Solutions were poured in 30 mL of water and resulting colloidal suspensions were transferred into dialysis tube (molecular weight cut off, 6–8000 Da) and dialyzed against water for 3 days prior to lyophilization.

### 2.6. Polymer Characterization

^1^H, ^19^F and ^13^C NMR spectra were recorded in CDCl_3_ or H_2_O-D_2_O (9:1) on a Bruker AV400 MHz (Billerica, MA, USA). Size exclusion chromatography (SEC) experiments were determined in chloroform using Shimadzu (Kyoto, Japan) LC-10AD pump with two Shodex GPC K-805 L columns (5 µm Mixte-C) using two PL aquagel-OH 40–30 columns (8 µm Mixte-C), at a concentration of 10 mg/mL. A Wyatt Technology (Santa Barbara, CA, USA) Optilab Rex interferometric refractometer was used as detector, and low polydispersity index polystyrene standards (3 × 10^4^ – 2 × 10^6^ g/mol) were used to calibrate the system. Grafting of fluorinated compounds was assessed by Raman spectroscopy using a Raman microscope LabRAMHR from Horiba Jobin Yvon (Longjumeau, France) equipped with a laser emitting at 633 nm. Water contact angle measurements were performed using the Drop Shape Analysis system on a Krüss goniometer (Villebon-sur-Yvette, France).

### 2.7. Preparation of Nanoparticles

Nanoparticles of perfluorobromide (PFOB) were prepared by an emulsion evaporation process adapted from [14]. Then 30 mg of PHA-F-PEG were dissolved into 2 mL of methylene chloride with 30 µL of PFOB. Organic phases were vortexed in 10 mL of 1.5% sodium cholate (*w*/*v*) aqueous solution for 1 min and then emulsified 1 min by sonication (Ultrasonic processor) at 20% of maximal power, over ice. Solvent was evaporated by magnetic stirring at room temperature for 3 h, at 300 rpm. Suspensions were purified to remove sodium cholate and putative non-encapsulated PFOB by ultracentrifugation for 45 min at 18,000× *g* at 4 °C. Pellets were resuspended in distilled water at the desired concentration and supernatants were analyzed by ^19^F NMR to ensure absence of residual PFOB.

### 2.8. Nanoparticles Characterization

Nanoparticles size and surface charge were measured by dynamic light scattering (DLS) and zeta potential (ZP) on a Malvern Zetasizer Nano ZS (Malvern, UK). Measurements were performed at 20 °C, at an angle of 173°. PHA-F-PEG nanoparticles morphology was investigated by transmission electron microscopy (Tecnai F20 microscope, FEI Company, Hillsboro, OR, USA). Samples were deposited on copper grids, dried at room temperature and then were colored with uracyl acetate (1 wt.%). 

### 2.9. ^1^H/^19^F MRI Imaging

Images of phantoms containing nanoparticles solutions were acquired on a 7-T small animal MRI scanner (Bruker, Ettlingen, Germany) using a double tuned, ^1^H/^19^F coil at Neurospin (CEA, Saclay, France). Sample solutions (at 30 mg°mL^−1^) were placed in 1 mL syringes, water and commercial PFOB were used as references. Images were acquired by cross sections of the syringes containing solutions to be analyzed. ^1^H MRI images were acquired for localization of the samples and ^19^F MRI images were acquired using a turbo spin-echo sequence, (RARE factor = 64, TE = 23.3 ms, TR = 4550 ms, number of average = 100, FOV = 0.2 × 0.2 mm, slice thickness = 8 mm, measurement time = 15 min 10 s).

## 3. Results and Discussion

### 3.1. PHAU Production

**Figure 1:**
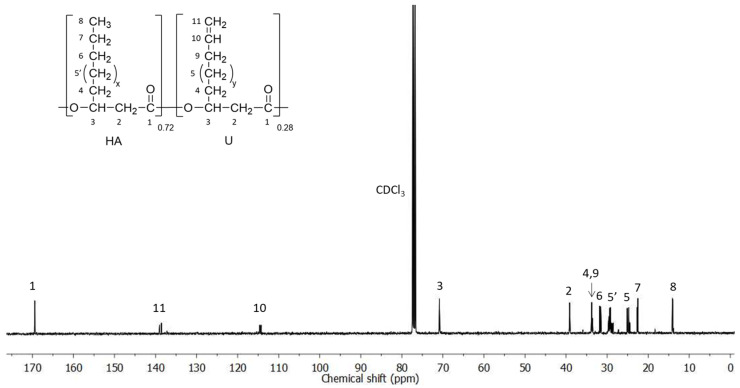
^13^C NMR spectra of PHAU. (x = 1 or 3 or 5; y = 2 or 4).

The ability of *Pseudomonas raguenesii* to produce unsaturated PHA (PHAU) was tested using coprah oil (5 g·L^−1^) that is a vegetable oil from coconut composed of more than 90% of saturated fatty acids, in mixture with 10-undecenoic acid (0.5 g·L^−1^) to induce PHAU production. After 72 h of culture, cells are harvested by centrifugation and PHAU is extracted from these freeze-dried pellets. After extraction and purification, PHAU production has a yield of 17% (*w*/*w*). The molar mass is 123,000 g.mol^−1^ with polydispersity index of 1.8. The ^13^C NMR spectrum of the polymer is shown in Figure 1 (HSQC spectrum is provided in the Supplementary Material, Figure S1). Chemical shifts of the C1 carbon (C=O) were detected at 169 ppm, that of C2 at 39 ppm and C3 at 71 ppm. C4 carbon of the side chain methylene groups is detected at 33 ppm. The chemical shifts at 14 ppm correspond to the terminal methyl groups (CH_3_) of the side chains and the chemical shifts observed between 22 ppm and 31 ppm correspond to the different methylene groups (CH_2_) of the side chains. The presence of a double bond is confirmed by the presence of peaks at 114 ppm and 139 ppm corresponding respectively to the methylene (=CH_2_) and methine (=CH–) groups of the double bond. The chemical shift at 34 ppm corresponds to the α-methylene groups of the double bond. 

### 3.2. PHA-g-(F; PEG) Synthesis from PHAU

**Figure 2:**
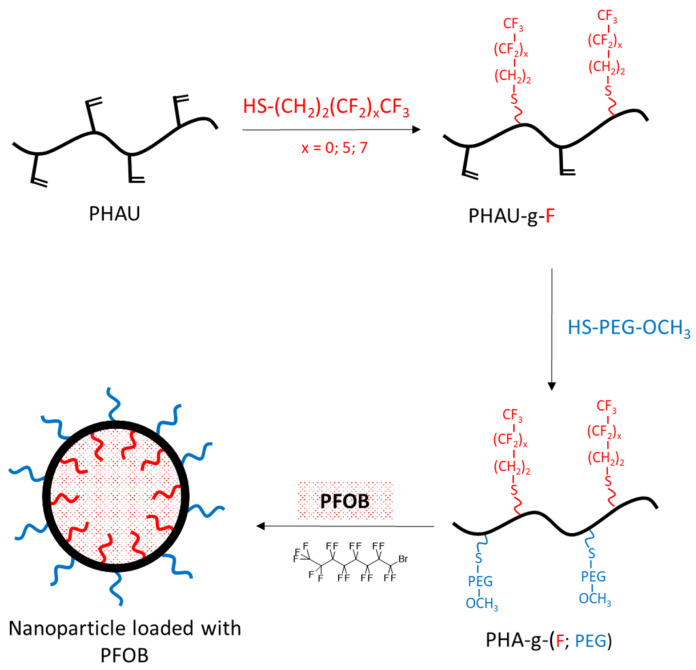
The synthetic route for the production of PFOB loaded nanocapsules.

Our strategy is to incorporate perfluoroalkyl side chains in the PHAU to enhance its tendency to self-assemble through the formation of fluorophilic interactions between perfluorinated side chains. Moreover, PEG grafting onto PHAU was performed to generate stealth material against the immune systems. The interest in preparing these amphiphilic copolymers that are both covalently grafted by PEG and fluorinated groups is 2-fold. It allows one both to increase the PFOB encapsulation and to avoid aggregation of the nanoparticles. The synthetic route to PHA-g-(F; PEG) is given in Figure 2. Different fluorinated thiols were grafted on PHAU (Table 1) in order to evaluate the importance of the length of the grafted moiety on the signal detected by MRI. The molar ratio of thiol was adjusted so as to functionalize only a part of the available double bonds because the other part will be further used to graft PEG on the side chains of PHAU.

**Figure 3:**
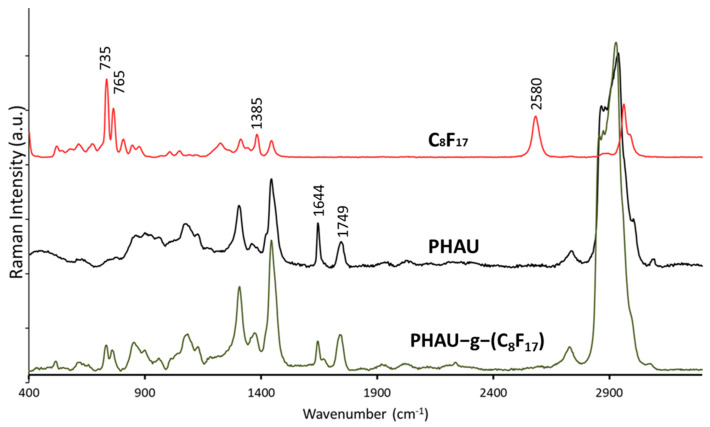
Raman spectra of C_8_F_17_, PHAU and PHAU-g-C_8_F_17_.

Raman spectrum (Figure 3) of PHAU-g-(C_8_F_17_) clearly showed the presence of fluorinated groups by the appearance of characteristic bands at 735 and 765 cm^−1^ assigned to symmetric CF_2_ stretching and symmetrical vibration involving the trifluoromethyl group CF_3_ respectively. The total disappearance of the band at 2580 cm^−1^ assigned to the SH group of the perfluorodecane-1-thiol showed that the thiol-ene reaction is quantitative. Moreover, the absence of the characteristic disulfide bond signal, usually observed around 500–550 cm^−1^, confirms the correct grafting. The value equal to the ratio of integration of the ester absorption band at 1749 cm^−1^, characteristic of the ester in the PHAU backbone and the double bond at 1644 cm^−1^ assigned to the unsaturated group, were determined before and after the thiol-ene reaction. The results showed that 41% of the initial double bonds were involved during the thiol grafting which was in agreement with the results obtained by ^1^H NMR.

**Figure 4:**
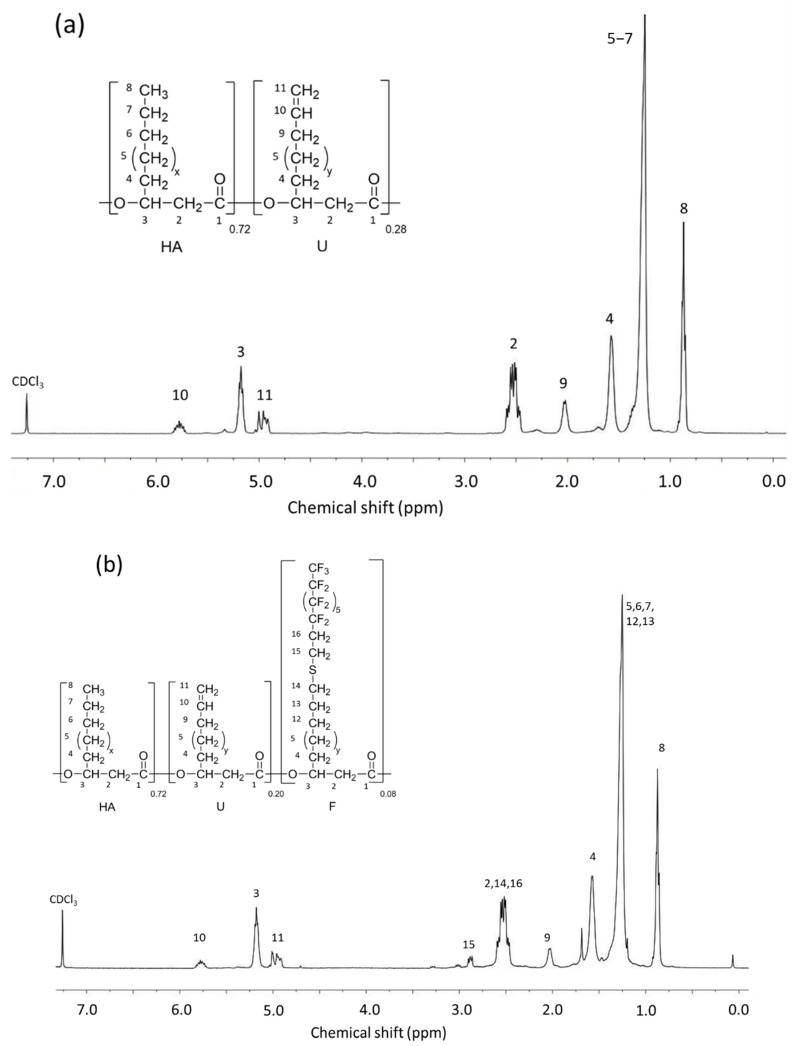
^1^H NMR spectra in CDCl_3_. (**a**) PHAU; (**b**) PHAU-g-C_8_F_17_. (x: 1,3,5; y: 2,4).

Initial PHAU was characterized by ^1^H NMR (Figure 4) to determine the percentage of double bonds by integrating protons corresponding to the methine peak (3) at 5.2 ppm and the signal relating to the terminal unsaturation group of side chain (10) at 5.7 ppm. PHAU is a statistical copolymer containing saturated and unsaturated side chains in the proportion 0.72/0.28 respectively. Perfluorodecane-1-thiol was grafted and the PHAU-g-(C_8_F_17_) was analyzed by ^1^H NMR (^1^H and ^19^F spectra of copolymers are provided in the Supplementary Materials). Signals relating to the CH_2_ methylene (15), characteristic of fluorinated groups, appeared at 2.8 ppm and indicated the success of the grafting. The conversion percentages of the double bonds after grafting were calculated by comparing the integrations of the protons corresponding to the CH peak (3) of the PHAU at 5.2 ppm, with respect to the signal of terminal unsaturation side chain (10) at 5.7 ppm.

**Figure 5:**
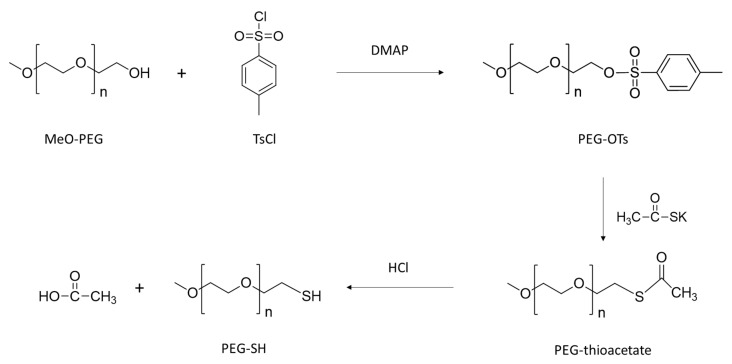
Synthesis of PEG-SH.

**Figure 6:**
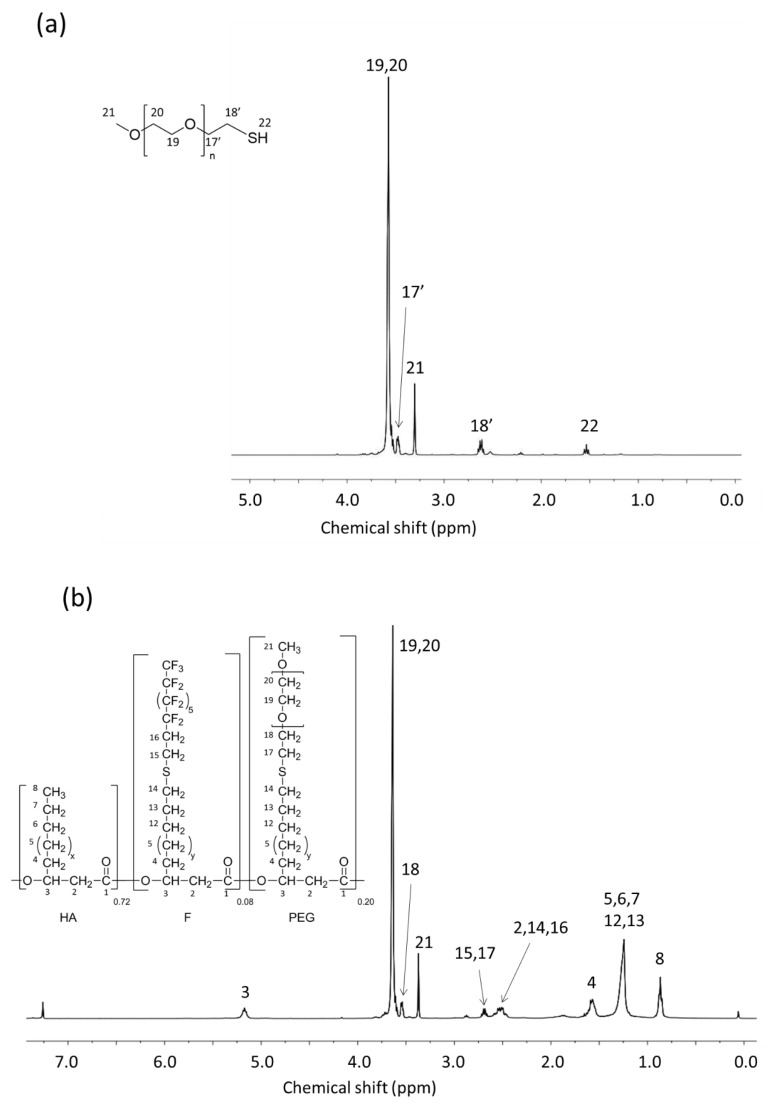
^1^H NMR spectra in CDCl_3_. (**a**) PEG_550_-SH; (**b**) PHA-g-(C_8_F_17_; PEG).

PEG550-SH was prepared in three steps according to a method described elsewhere [46] (Figure 5). Thiolated PEG was characterized by ^1^H NMR (Figure 6a) and the presence of signals at 2.60 and 1.55 ppm attested that the reaction is quantitative. In a second step PEG methyl ether thiol was grafted using the thiol-ene reaction to prepare PHA-g-(C_8_F_17_; PEG). Two peaks are observed at 3.6 and 3.3 ppm that are assigned to the CH= (19, 20) and CH_3_ (21) methyl terminal groups of the PEG confirming the success of the grafting (Figure 4b). The total disappearance of the peaks at 5.7 ppm (10) and 4.9 ppm (11) showed that there are no more unsaturated groups in the PHA. The compositions of the different copolymers determined by NMR are listed in Table 2.

The water contact angle of solvent cast PHAU film was found to be 96.2°, which was in agreement with literature, whereas the contact angle was about 132.5° for PHA_72_U_20_-g-(C_8_F_17_)_8_. This difference can be explained by the presence of very hydrophobic fluorinated groups. The water contact angle decreased to 106.3° after PEG grafting but it remained higher than the value of the initial PHA, attesting to the global hydrophobic character of the copolymer.

### 3.3. Synthesis and Characterization of the Nanoparticles

**Figure 7:**
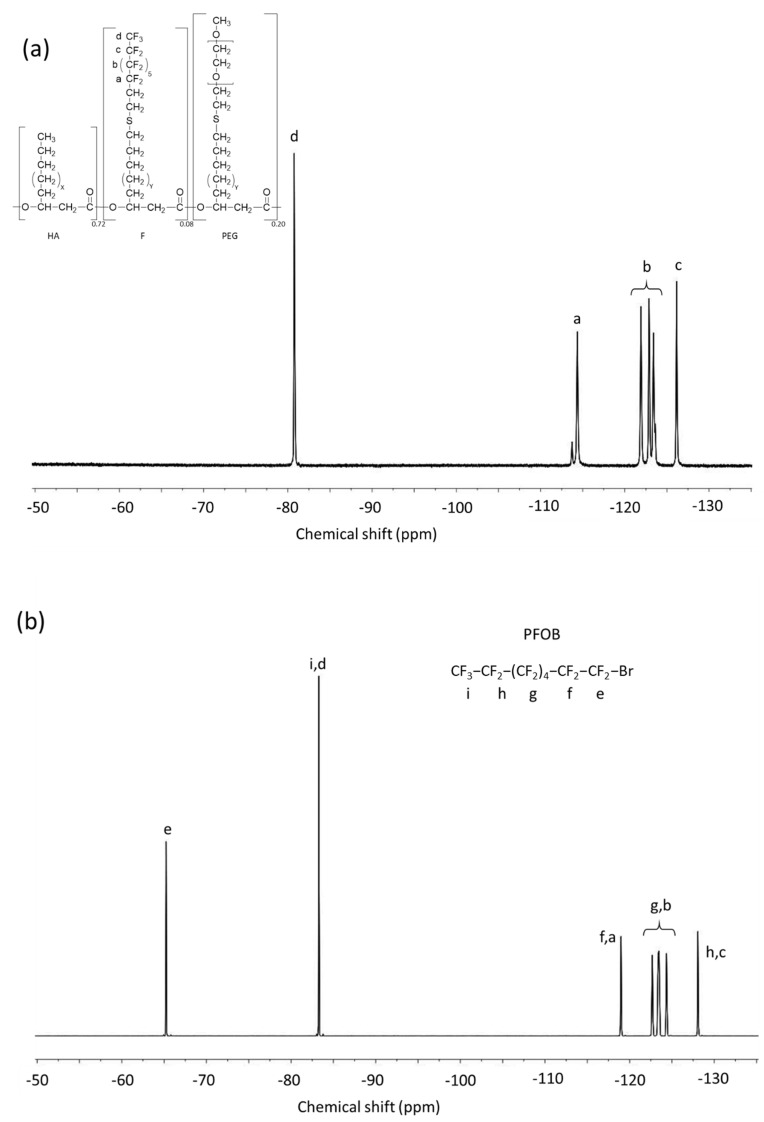
^19^F NMR spectra. (**a**) PHA−g−(F; PEG) in CDCl_3_; (**b**) Nanocapsules containing PFOB in H_2_O-D_2_O (9:1).

PHAU-g-(F; PEG) PFOB nanoparticles were prepared by an emulsion evaporation method. In these conditions, the graft copolymers will adopt a specific conformation in the aqueous medium. The perfluorinated side chains may orientate towards the PFOB core whereas the hydrophilic PEG grafts may orientate towards the aqueous medium. The ^19^F NMR spectrum of the nanocapsules reveals peaks consistent with the presence of three different containing sites in the fluorinated moieties. The peak at −65 ppm originates from CF_2_ near the bromide atom, the one at −84 ppm from CF_3_ fluorine and the peaks between −118 and −127 ppm from CF_2_ fluorine of the PFOB. Although the peaks characteristic of the PHAU-g-(F; PEG) were clearly distinguished on the spectrum of the copolymer alone, they were hidden by the PFOB ones (Figure 7).

**Figure 8:**
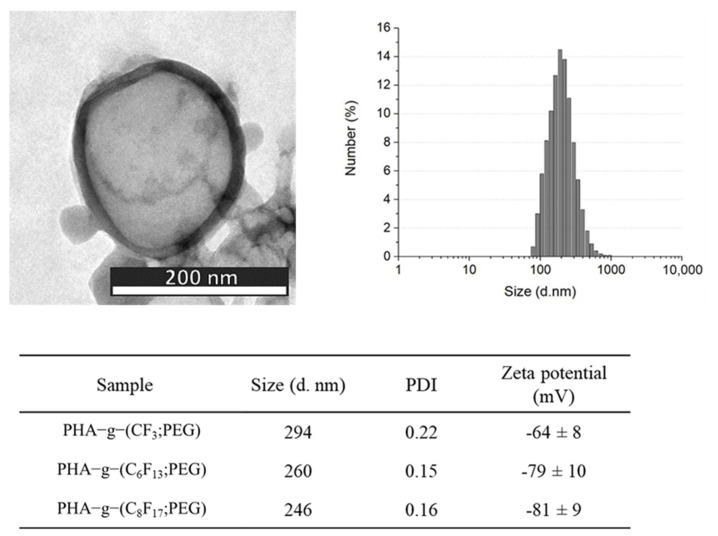
Characterization of nanocapsules by TEM and DLS.

Negative staining improved the TEM image contrast and allowed the visualization of the inner core in PFOB in light gray whereas the polymeric shell seemed darker, which confirmed the core-shell structure (Figure 8). Images show spherical objects with size in good agreement with the diameter measured by dynamic light scattering DLS. Smooth surfaces are observed for all particles. DLS measurements showed that the average size of PHA-g-(F; PEG) PFOB nanoparticles increased from 246 nm for the perfluorodecane graft to 294 nm for the trifluoropropane graft. The increase of the length of the fluorinated side chains certainly improves the interactions between these hydrophobic moieties. A wider distribution of sizes was also observed. All samples present very negative zeta potential regardless of the length of the perfluorinated groups, resulting in a very good stability in aqueous medium with a low tendency to aggregation [48].

### 3.4. In Vitro ^19^F NMR

**Figure 9:**
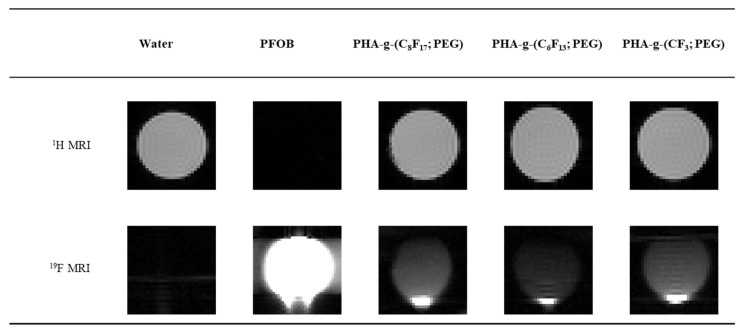
In vitro ^1^H MRI and ^19^F MRI phantom images of the different nanocapsules containing PFOB. (RARE factor = 64; TE = 23.3 ms, TR = 4550 ms; scan time = 15 min 10 s).

Phantom MRI studies were performed to evaluate in vitro the contrast properties of PFOB nanocapsules from different PHA-g-(F; PEG). These nanocapsules were dissolved in distilled water and different acquisition times were evaluated: 1 min 31 s, 15 min 10 s, 1 h 16 min and 10 h. It turns out that from 15 min 10 s, nanocapsules solutions are distinctly visible and as detectable as after 10 h of acquisition. This relatively short analysis time is very encouraging to consider a further clinical application even though fluorine concentration would probably by lower after an injection in vivo. The ^1^H MRI and ^19^F MRI images thus acquired in 15 min were presented in Figure 9. A more intense signal is observed at the bottom of the images due to the nanocapsules sedimentation during the different acquisitions. One of the simple solutions to increase the visibility in MRI could be to increase the number of fluorine atoms per nanocapsule. Here, the number of F atoms varied from 3 to 17 per grafted fluorinated segment for the three imaged nanocapsules. The results obtained show that this is not a determining parameter because the particles are visible whatever the length of the grafted fluorinated segment. The CF_3_ is volatile, which can appear as a disadvantage during the grafting reactions and therefore it would be preferable to use C_6_F_13_ or C_8_F_17_. The preliminary results showed that these PFOB nanocapsules could be used as contrast agents for MRI.

## 4. Conclusions

An efficient and reproducible method for the synthesis of copolymers of amphiphilic PHA-g-(F;PEG) has been developed by two successive thiol-ene reactions. These copolymers will be used for the encapsulation of PFOB known to be a contrast agent for MRI. The graft copolymers based on unsaturated PHAs produced by marine bacteria have been functionalized with on one hand perfluorinated groups to promote interactions with PFOB and on the other hand with PEG to improve hydrophilicity and escape the phagocytic cells of the immune system. The molar composition of the copolymer is strictly controlled due to the efficiency of the thiol-ene addition. These nanocapsules based on PHA-g-(F;PEG) would constitute a new generation of biocompatible therapeutic nanovectors that can be tracked by MRI thanks to their contrast agents capabilities.

## Figures and Tables

**Table 1:** Chemical structures of the different perfluorinated thiols.

Fluorinated Thiol	Chemical Structure	Abbreviation
Trifluoropropane-1-thiol	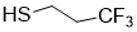	CF_3_
Perfluorooctane-1-thiol	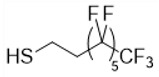	C_6_F_13_
Perfluorodecane-1-thiol	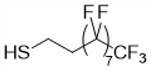	C_8_F_17_

**Table 2:** Water contact angles and compositions of the different copolymers.

Copolymers	PEG (%) ^1^	C_8_F_17_ (%) ^1^	Contact Angle (°)
PHAU	-	-	96.2
PHAU-g-(C_8_F_17_)	-	8	132.5
PHA-g-(C_8_F_17_; PEG)	20	8	106.3

^1^ determined by ^1^H NMR.

## Data Availability

Not applicable.

## Supplementary Materials

The following are available online at https://www.mdpi.com/article/10.3390/bioengineering8090121/s1, Figure S1: HSQC spectrum of PHAU in CDCl_3_, Figure S2: ^1^H NMR spectra of C_8_F_17_ (red), PHAU (black), and PHAU-g-(C_8_F_17_) (green) in CDCl_3_, Figure S3: ^19^F NMR spectra of PHAU-g-(C_8_F_17_) in CDCl_3_, Figure S4: ^1^H NMR spectra of C_6_F_13_ (red), PHAU (black), and PHAU-g-(C_6_F_13_) (green) in CDCl_3_, Figure S5: ^19^F NMR spectra of PHAU-g-(C_6_F_13_) in CDCl_3_, Figure S6: ^1^H NMR spectra of CF3 (red), PHAU (black), and PHAU-g-(CF_3_) (green) in CDCl_3_, Figure S7: ^19^F NMR spectra of PHAU-g-(CF_3_) in CDCl_3_.
