# Supplementary material for: Amphiphilic and Perfluorinated Poly(3-Hydroxyalkanoate) Nanocapsules for 19F Magnetic Resonance Imaging
Source: Bioengineering (Basel). 2021 Sep 9;8(9):121. doi: 10.3390/bioengineering8090121 (PMC8466264; doi:10.3390/bioengineering8090121)
Supplement: Supplementary file 1 [file bioengineering-08-00121-s001.zip › bioengineering-1292453-supplementary.pdf]

bioengineering

MDPI

Supplementary Materials

Amphiphilic and Perfluorinated Poly(3-Hydroxyalkanoate) Nanocapsules for ^{19}F Magnetic Resonance Imaging

Marion Le Gal [1,2], Estelle Renard [2], Christelle Simon-Colin [1], Benoit Larrat [3] and Valérie Langlois [2,*]

[1] Laboratoire de Microbiologie des Environnements Extrêmes, CNRS, Ifremer, University Brest, F-29280 Plouzané, France; mlegal@ifremer.fr (M.L.G); christelle.simon.colin@ifremer.fr (C.S.-C.)
[2] University Paris Est Creteil, CNRS, ICMPE, F-94010 Creteil, France; renard@icmpe.cnrs.fr
[3] Université Paris-Saclay, CEA, CNRS, NeuroSpin, 91191 Gif-sur-Yvette, France; benoit.larrat@cea.fr
* Correspondence: langlois@u-pec.fr

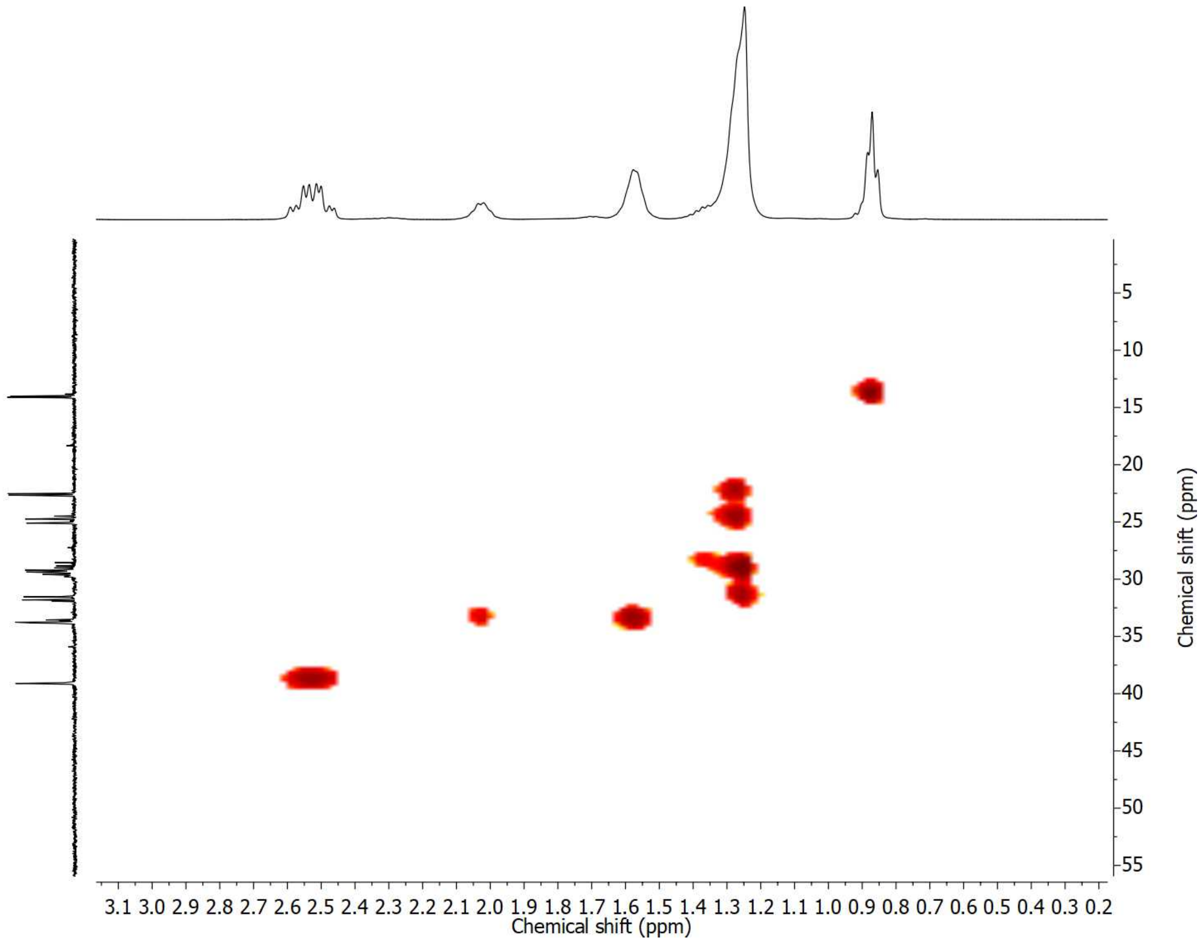

Figure S1. HSQC spectrum of PHAU in $CDCl_3$.

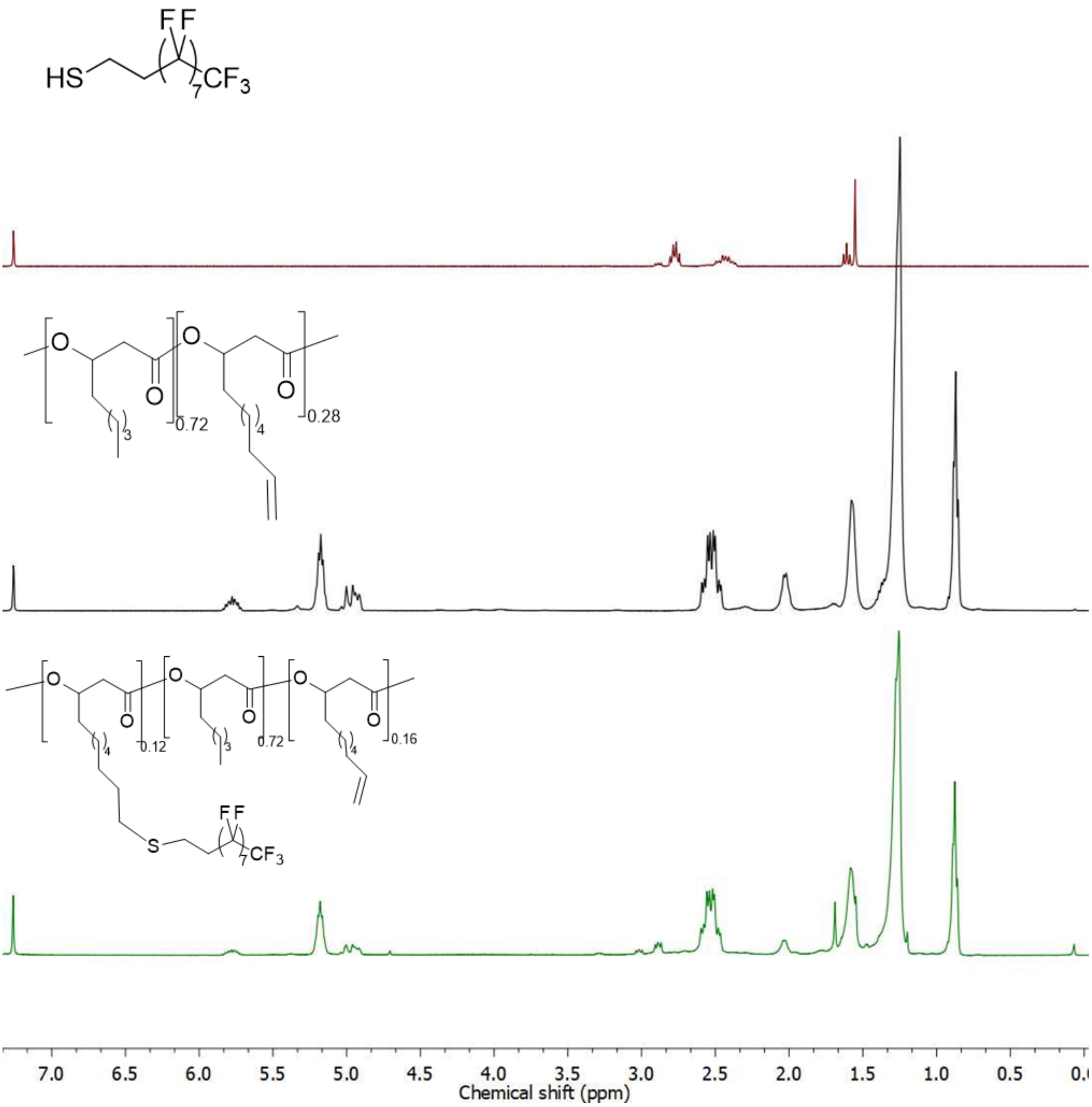

Figure S2. ^{1}H NMR spectra of C_8F_{17} (red), PHAU (black), and PHAU-g-(C_8F_{17}) (green) in $CDCl_3$.

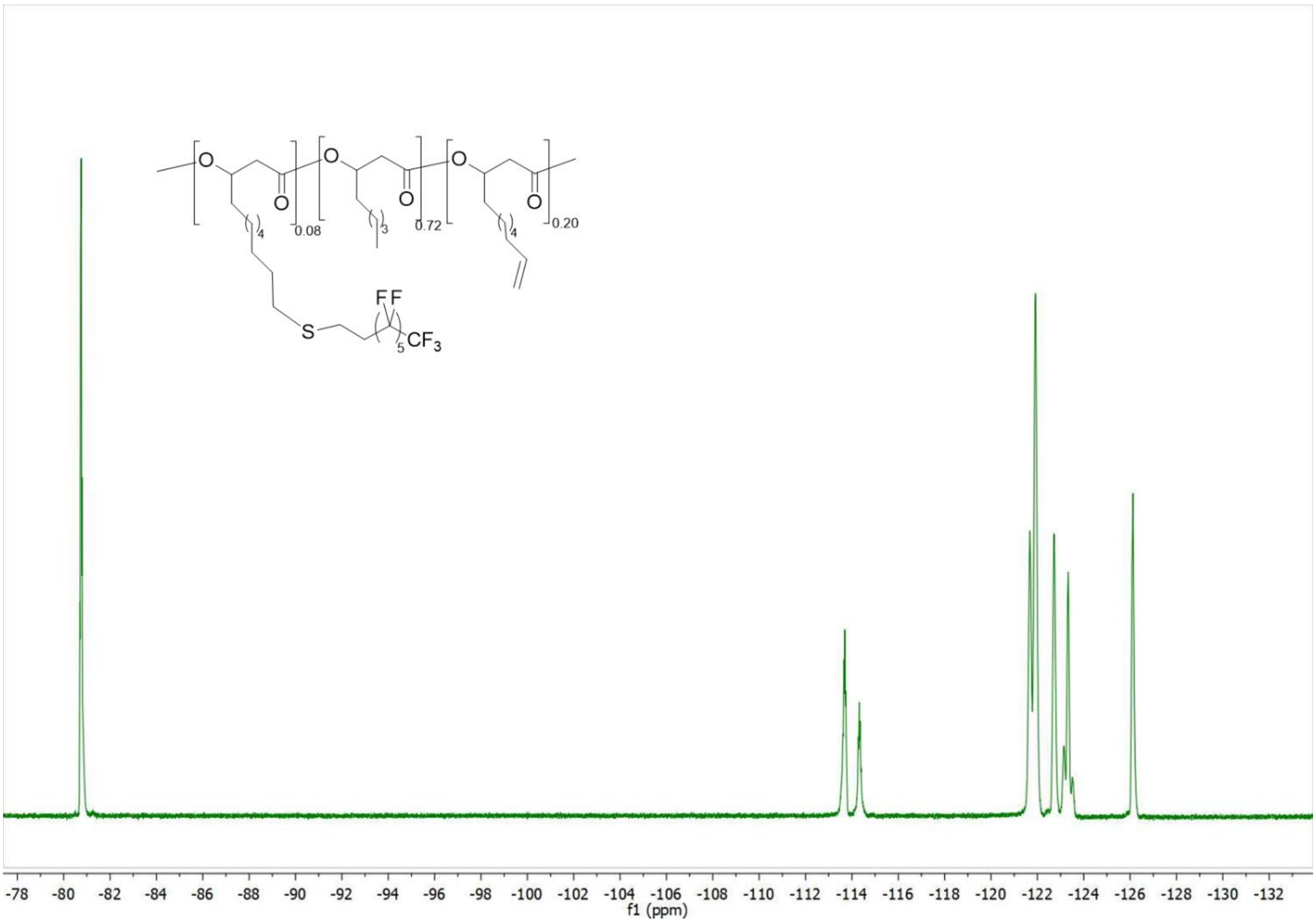

Figure S3. ^{19}F NMR spectra of PHAU-g-(C_8F_{17}) in $CDCl_3$.

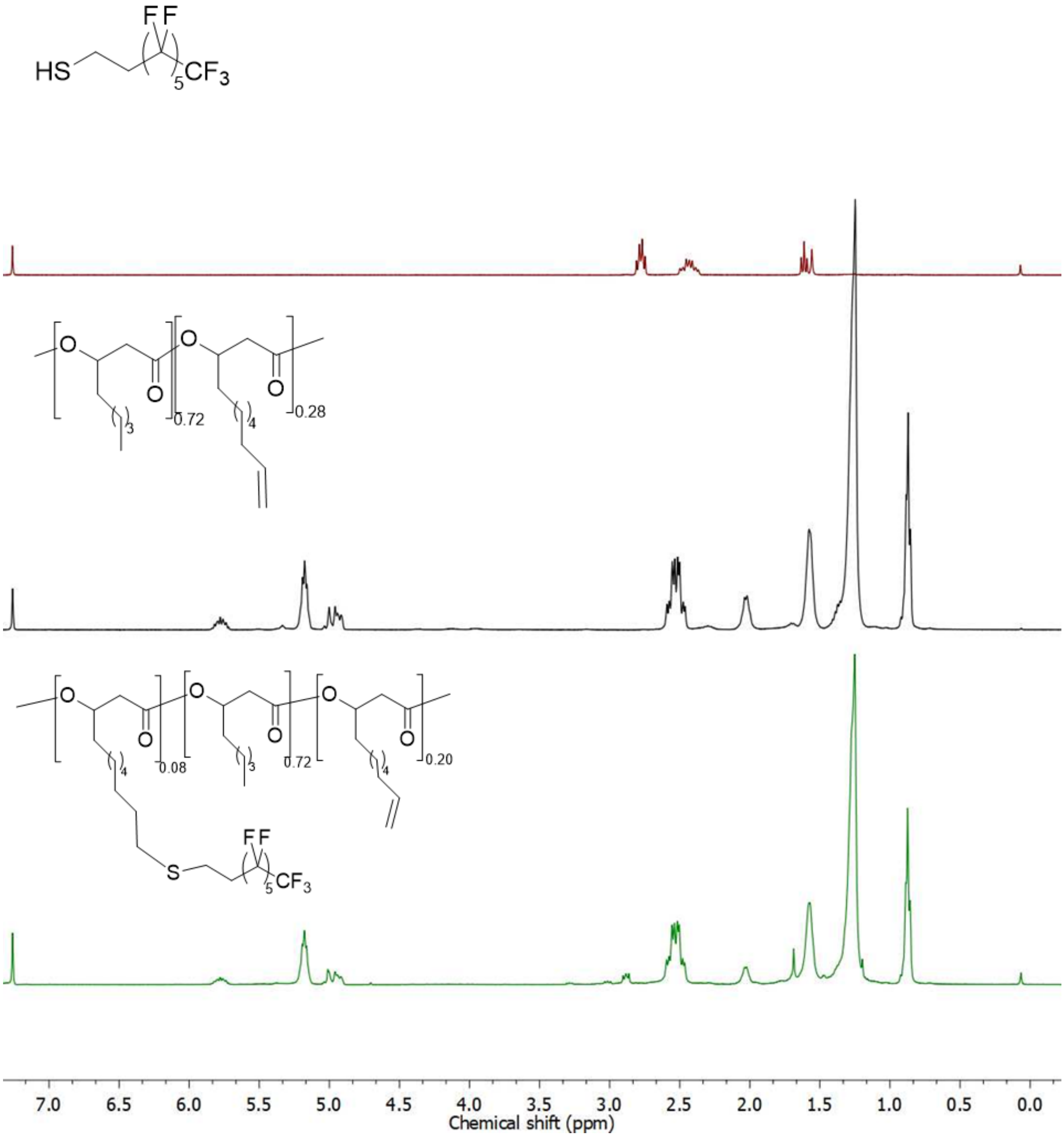

Figure S4. ^{1}H NMR spectra of C_6F_{13} (red), PHAU (black), and PHAU-g-(C_6F_{13}) (green) in $CDCl_3$.

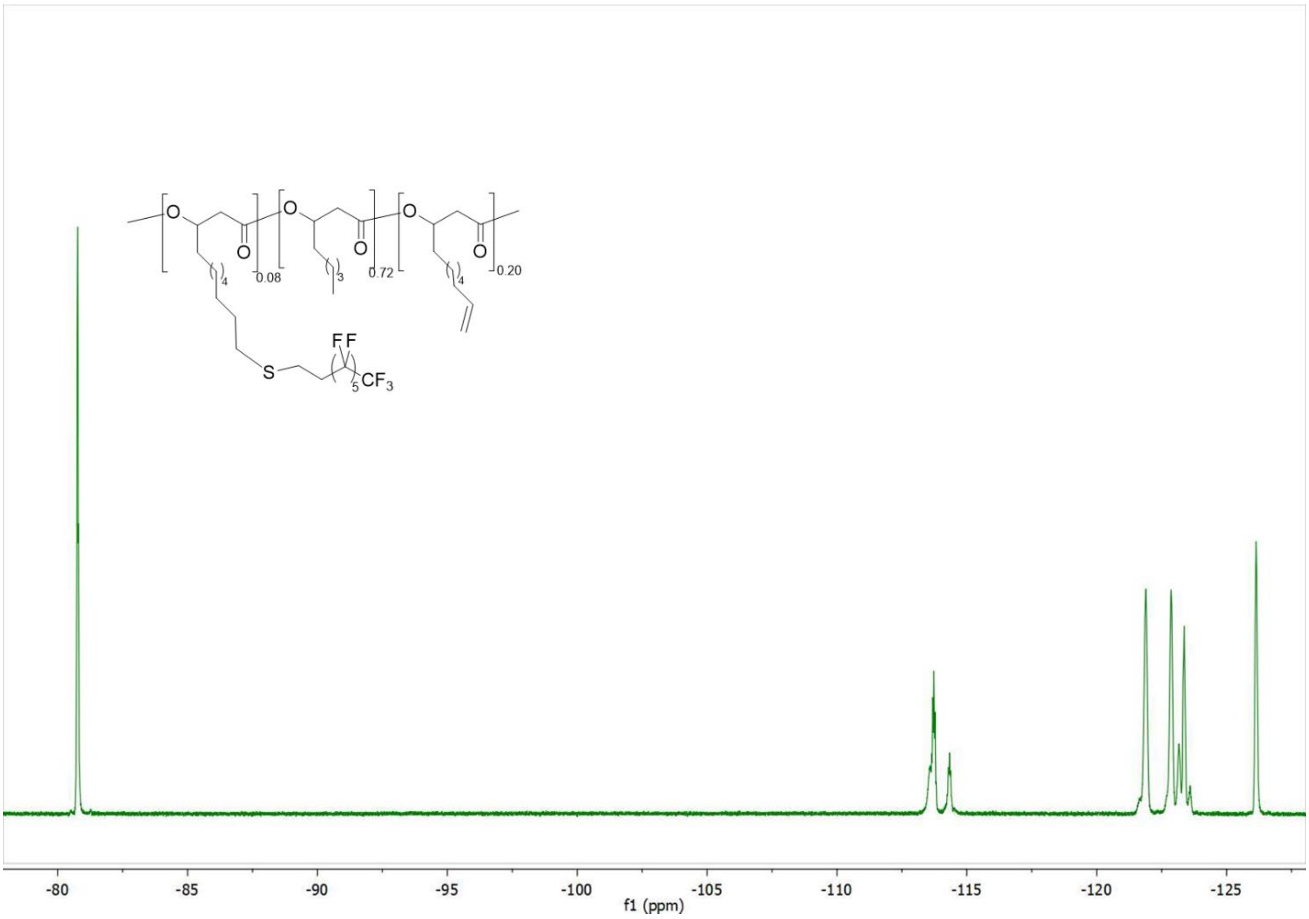

Figure S5. ^{19}F NMR spectra of PHAU-g-(C_6F_{13}) in $CDCl_3$.

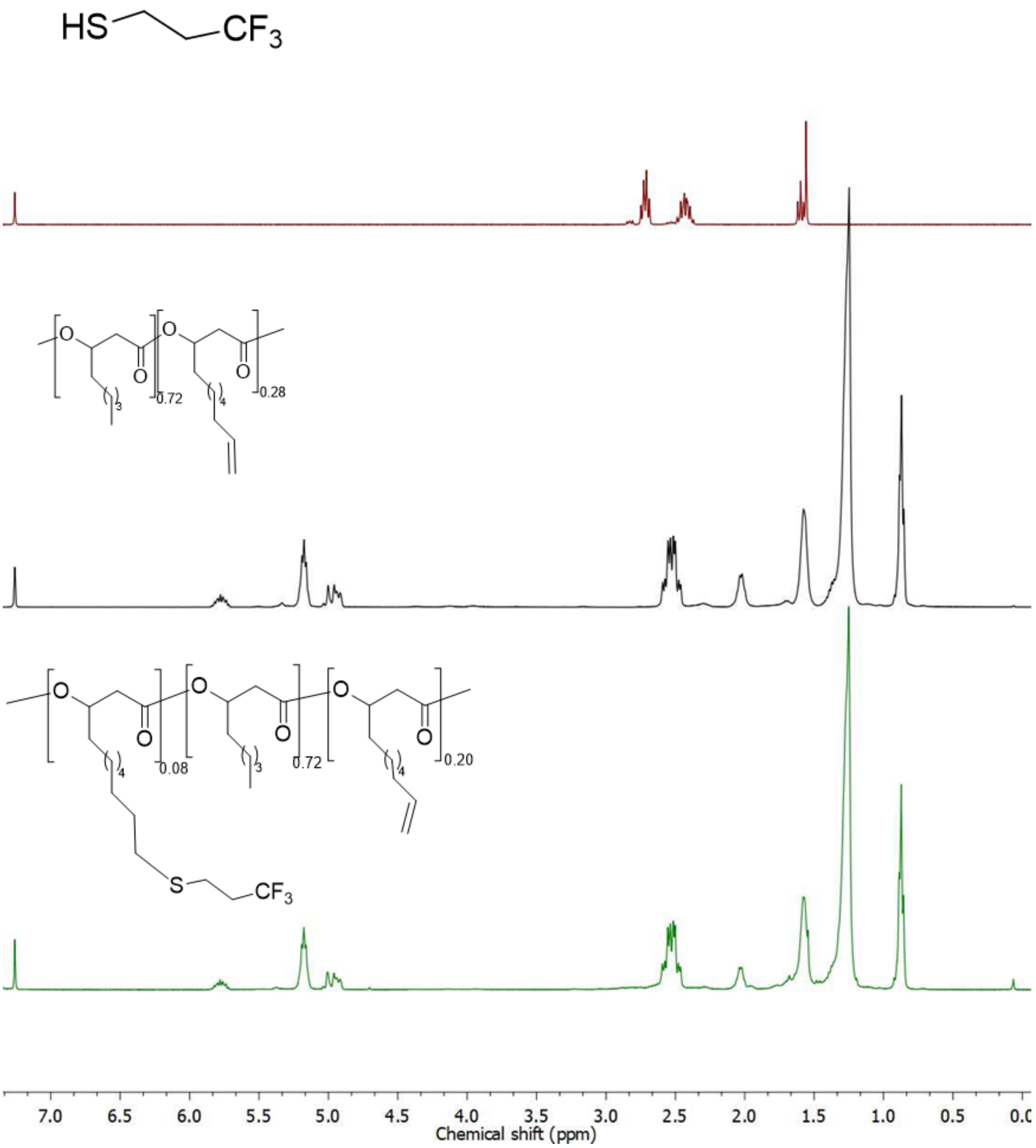

Figure S6. ^{1}H NMR spectra of CF_3 (red), PHAU (black), and PHAU-g-(CF_3) (green) in $CDCl_3$.

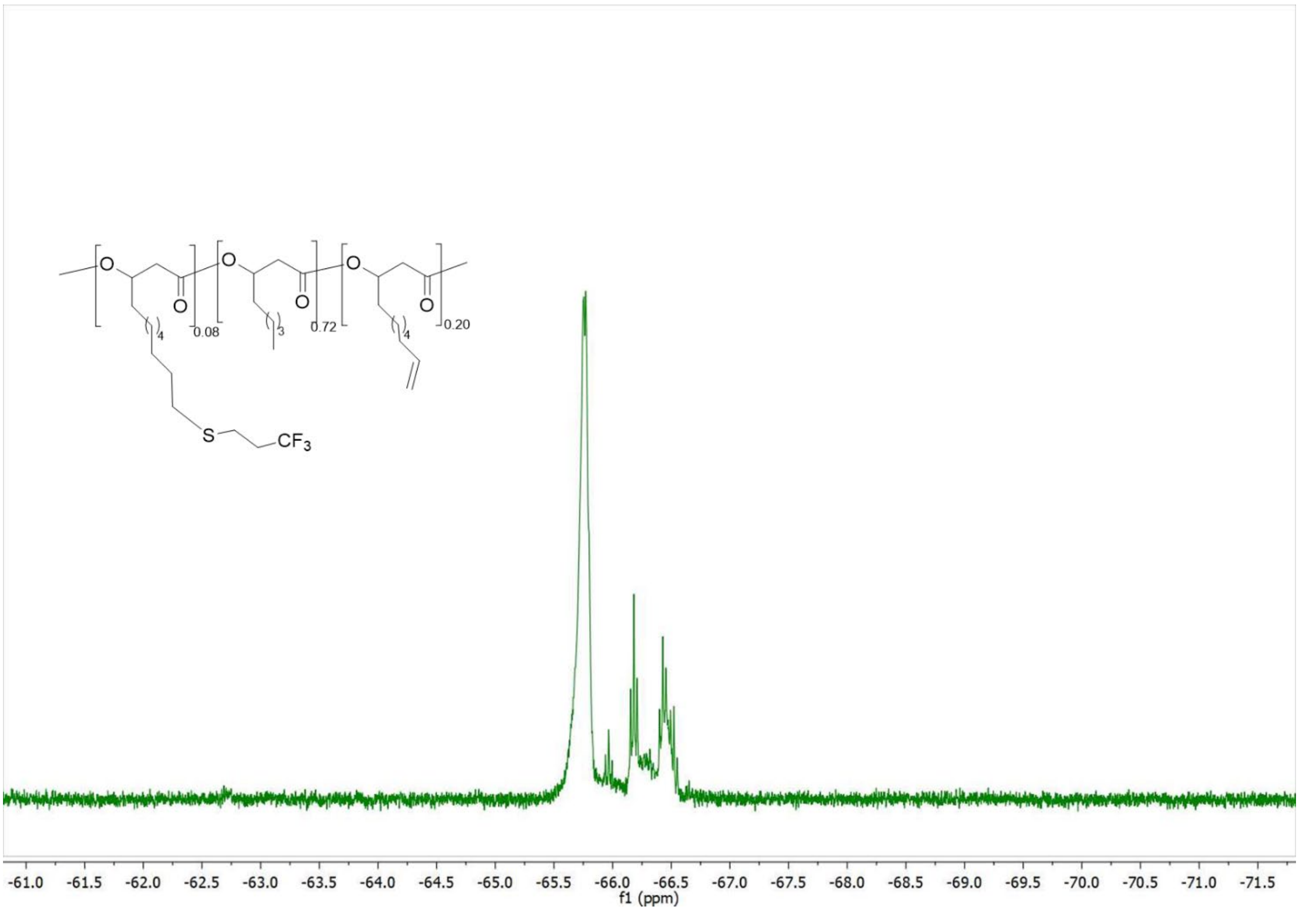

Figure S7. ^{19}F NMR spectra of PHAU-g-(CF_3) in $CDCl_3$.
